# Dual chirped microcomb based parallel ranging at megapixel-line rates

**DOI:** 10.1038/s41467-022-30542-x

**Published:** 2022-06-07

**Authors:** Anton Lukashchuk, Johann Riemensberger, Maxim Karpov, Junqiu Liu, Tobias J. Kippenberg

**Affiliations:** Laboratory of Photonics and Quantum Measurements (LPQM), Swiss Federal Institute of Technology Lausanne (EPFL), CH-1015 Lausanne, Switzerland

**Keywords:** Optical sensors, Nonlinear optics, Solitons, Imaging and sensing

## Abstract

Laser-based ranging (LiDAR) - already ubiquitously used in industrial monitoring, atmospheric dynamics, or geodesy - is a key sensor technology. Coherent laser ranging, in contrast to time-of-flight approaches, is immune to ambient light, operates continuous-wave allowing higher average powers, and yields simultaneous velocity and distance information. State-of-the-art coherent single laser-detector architectures reach hundreds of kilopixel per second sampling rates, while emerging applications - autonomous driving, robotics, and augmented reality - mandate megapixel per second point sampling to support real-time video-rate imaging. Yet, such rates of coherent LiDAR have not been demonstrated. Recent advances in photonic chip-based microcombs provide a route to higher acquisition speeds via parallelization but require separation of individual channels at the detector side, increasing photonic integration complexity. Here we overcome the challenge and report a hardware-efficient swept dual-soliton microcomb technique that achieves coherent ranging and velocimetry at megapixel per second line scan measurement rates with up to 64 optical channels. Multiheterodyning two synchronously frequency-modulated microcombs yields distance and velocity information of all individual ranging channels on a single receiver alleviating the need for individual separation, detection, and digitization. The reported LiDAR implementation is compatible with photonic integration and demonstrates the significant advantages of acquisition speed afforded by the convergence of optical telecommunication and metrology technologies.

## Abstract (web summary)

Photonic integrated systems can be harnessed for fast and efficient optical telecommunication and metrology technologies. Here the authors develop a dual-soliton microcomb technique for massively parallel coherent laser ranging that requires only a single laser and a single photoreceiver.

## Introduction

Three-dimensional (3D) imaging based on lasers is ubiquitously used in numerous applications, ranging from airborne imaging for the cartography of geological sites^1^ and urban planning to satellite-based applications in space. A recent surge in autonomous driving^2^ and drone technology^3^ drives the development of even more sophisticated laser ranging systems. LiDAR maintains excellent angular resolution at long range and works reliably in a variety of weather, illumination and target conditions that impede direct camera imaging. While most commercial implementations of LiDAR employ incoherent detection of the intensity of reflected light, coherent detection of the back-reflected signal using a copy of the transmitted optical waveform^4–6^ is intrinsically resilient to crosstalk and interference from ambient sunlight detection^7^. Furthermore, it achieves high depth resolution dependent on chirp excursion without the need for high-bandwidth electronics^8,9^ and gives both distance and velocity (via the Doppler effect) for each pixel^10^ facilitating object classification.

One challenge to harvesting the inherent advantages of coherent or frequency-modulated continuous-wave (FMCW) LiDAR for 3D imaging is to overcome the frame-rate acquisition bottleneck that is imposed by tunable diode laser sources that trade-off tunability versus linewidth^11^ and artificial Doppler broadening due to the mechanical tilt motion of the mirrors, which necessitates inertia-free scanning solutions. A video frame rate (30 Hz) with 600 × 300 pixel images requires more than 5 megapixel/second measurement rates. Such large frame rates cannot be attained by increasing measurement speed due to limitations imposed by mechanical scanning, and pixel dwell time, i.e., signal-to-noise ratio. A manifold of solutions for inertia-free scanning based on photonic switching networks^12^, focal plane arrays^9^, spectrally encoded spatial scanning with broadband^13–15^ or frequency swept light sources^16,17^, or optical phased arrays^18^ have been implemented. Yet to date, megapixel rate coherent LiDAR has not been demonstrated.

Parallel detection architectures can further increase the acquisition rates. In time-of-flight LiDAR, it supports the operation of up to 128 channels. Recently, dissipative Kerr solitons (DKS)^19^—coherent frequency combs generated in a continuous wave-driven Kerr nonlinear microresonator—showed the parallelization of FMCW laser into multiple optical channels (although electro-optical combs are equally suitable^20,21^). DKS can faithfully transfer the time-frequency characteristics of an FMCW pump laser to all comb teeth^22^ at modulation speeds up to 10 MHz with a mode spacing of 100 GHz (supported by commercial multiplexers). The large comb spacing facilitates the spatial separation of the comb teeth with diffractive optics. Each tooth can independently and simultaneously measure the distance and velocity in a parallel fashion. However, the number of optical channels employed determines the number of balanced photo-receivers, electrical amplifiers and analog-to-digital converters. Custom, large-area silicon photonic solutions^9,12^ and dense wavelength-division multiplexers would be required to unlock the potential of massively parallel FMCW ranging.

Here, we demonstrate a hardware-efficient massively parallel coherent FMCW LiDAR based on multiheterodyne mixing of two photonic chip-based soliton microcombs on a single coherent photoreceiver, enabling bona fide 5.6 megapixels per second measurement rates, with more than 64 simultaneous channels.

## Results

### Concept of hardware-efficient megapixel coherent ranging

Our approach is a swept frequency version of multiheterodyne detection^23^ of optical frequency combs, commonly referred to as dual-comb spectroscopy. This technique has attained widespread attention and application in (nonlinear) optical and THz spectroscopy^24–26^, optical microscopy^27,28^, distance measurement^29–31^, two-way time-frequency transfer^32^, microwave photonics^33^, multi-dimensional spectroscopy^34^, coherent anti-Stokes Raman imaging^35^, and recently demonstrated spectrally interleaved broadband spectroscopy with frequency swept electro-optical dual-comb^36,37^. This method decodes all individual low frequency (MHz bandwidth) channels using a single high speed (GHz) coherent ‘intradyne’ detector by radio-frequency multiplexing. These detectors are nowadays widespread in data centers^38^; for example, a recently introduced silicon photonics-based coherent optical pluggable transceiver 400ZR supports 64 GBaud modulation speeds^39^, which would constitute an off-the-shelf component solution for a chip-scale FMCW LiDAR.

**Fig. 1:**
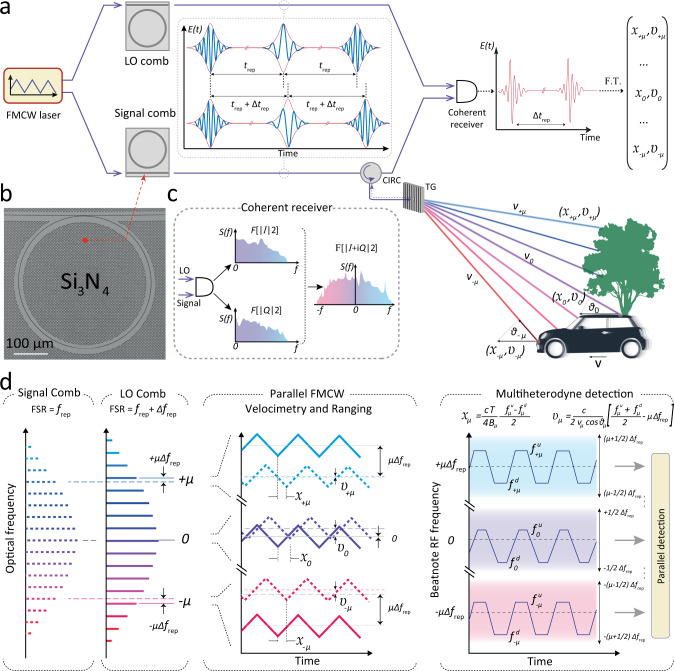
Multiheterodyne parallelization of coherent laser ranging. **a** Architecture of the multiheterodyne parallel FMCW LiDAR. A single pump laser with triangular frequency modulation drives two distinct optical microresonators with slightly different radii, which serve as signal and local oscillator (LO) in the experiment. The signal comb is spatially dispersed over the target area using diffractive optics. Each signal comb tooth *μ* (with an optical frequency *ν*_*μ*_) represents an independent FMCW ranging channel measuring distance *x*_*μ*_ and velocity *υ*_*μ*_. All channels are simultaneously superimposed with the LO comb on a coherent receiver. The interferogram is processed via short-time Fourier transform analysis to retrieve distances *x*_*μ*_ and velocities *υ*_*μ*_. **b** Electron microscope picture of 228.43 *μ*m Si_3_N_4_ microring resonator. **c** The complex RF spectrum is retrieved by phase diversity detection and Fourier transform. **d** Principle of multiheterodyne ranging and velocimetry. The Signal and LO combs have repetition rates *f*_rep_ of 98.90 GHz and 99.39 GHz, respectively. The reflected signal comb light is time delayed and frequency shifted due to the Doppler effect. Beat notes of consecutive comb tooth pairs are spaced 490 MHz in the RF spectrum. Triangular frequency modulation maps the distance of target objects to two RF beat notes, ${f}_{\mu }^{{{{{{{{\rm{d}}}}}}}}}$ and ${f}_{\mu }^{{{{{{{{\rm{u}}}}}}}}}$, spaced around the center frequency of the multiheterodyne channel *μ* ⋅ *f*_rep_ offset by the Doppler shift caused by the relative velocity of LiDAR transmitter and target.

In our experiments (cf. Fig. 1a), we utilized a single highly coherent FMCW laser that was amplified, split and coupled into two size-mismatched photonic chip-based integrated Si_3_N_4_^40^ microring resonators (cf. Fig. 1b) driving two dissipative Kerr solitons^41^. Fast frequency tuning of the pump laser within the soliton existence range retains the DKS state in both resonators^22^. The rapid frequency modulation is encoded onto the carrier-envelope frequency *f*_ceo_ of the pulse while the pulse repetition rate *f*_rep_ remains almost constant. In the frequency domain (cf. Fig. 1d), we obtain two soliton microcombs with slightly different comb line spacing Δ*f*_rep_ where each comb line inherits the pump laser frequency modulation. Multiheterodyne mixing of the reflected signal comb with the local oscillator (LO) comb on a single coherent photoreceiver enables the reconstruction of the entire complex RF spectrum (cf. Fig. 1c, d), which contains the distance *x*_*μ*_ and velocity *υ*_*μ*_ information for each comb line *μ* simultaneously (*μ* denotes the relative mode number with respect to the pump laser mode).

In comparison to conventional FMCW LiDAR^10^, multiheterodyne detection modifies the formulas to calculate (*x*_*μ*_, *υ*_*μ*_) from the beat notes ${f}_{\mu }^{{{{{{{{\rm{u}}}}}}}}},{f}_{\mu }^{{{{{{{{\rm{d}}}}}}}}}$ measured during the up- and down-chirping of the FMCW laser because the intermediate frequency is no longer at the baseband. Instead, consecutive channels in the radio-frequency (RF) domain are separated by the difference in comb line spacing Δ*f*_rep_. To mitigate the degeneracy in optical detection between +*μ* and −*μ* comb lines located symmetric about the pump (*μ* = 0), we employ a phase diversity receiver architecture^42,43^ and measure both the in-phase (*I*) and quadrature (*Q*) components of the multiheterodyne beat note (cf. Fig. 1c). The Fourier transform of the complex field amplitude *I* + *iQ* distinguishes positive and negative frequencies in the multiheterodyne beat spectrum^44^. The deviation of the beat note pattern from *μ* ⋅ Δ*f*_rep_ determines the Doppler shift and non-zero detection distance translates into a splitting of the RF beat note

1 $$\begin{array}{l}{x}_{\mu }=\frac{cT}{4{B}_{\mu }}\cdot \frac{{f}_{\mu }^{{{{{{{{\rm{u}}}}}}}}}-{f}_{\mu }^{{{{{{{{\rm{d}}}}}}}}}}{2}\\ {v}_{\mu }=\frac{c}{2{\nu }_{\mu }cos{\theta }_{\mu }}\cdot \left[\frac{{f}_{\mu }^{{{{{{{{\rm{u}}}}}}}}}+{f}_{\mu }^{{{{{{{{\rm{d}}}}}}}}}}{2}-\mu {{\Delta }}{f}_{{{{{{{{\rm{rep}}}}}}}}}\right],\end{array}$$

where *υ*_*μ*_*cosθ*_*μ*_ is a projection of the target velocity along the optical path and *ν*_*μ*_ is the optical frequency of the *μ*-th comb line.

**Fig. 2:**
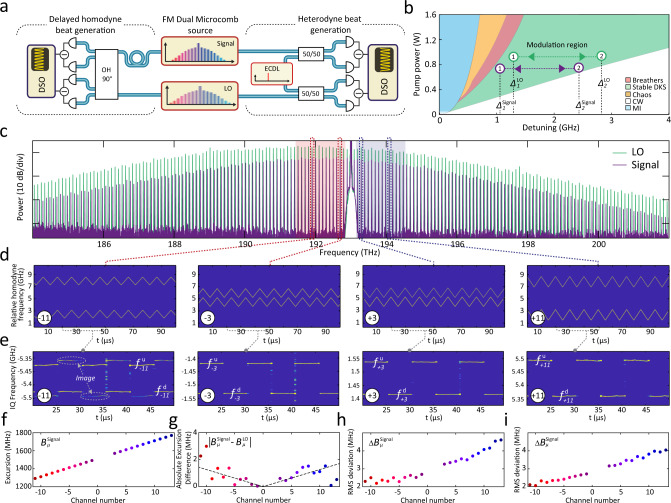
Coherent detection of multiple frequency modulated laser channels. **a** Optical setup for heterodyne and delayed homodyne beat note measurement. The signal microcomb is delayed and mixed in a 90^∘^ optical hybrid and superimposed with the local oscillator (LO) microcomb on a pair of balanced photoreceivers for delayed homodyne beat frequency generation. Alternatively, the tuning of individual comb line pairs is characterized by heterodyne mixing with an external cavity diode laser (ECDL). **b** Soliton microcomb stability chart. Optical resonances are thermally tuned to superimpose the laser-cavity detuning regions of stable soliton generation (green shaded). MI modulation instability, CW continuous-wave. **c** Optical spectra of signal and LO combs featuring a slight offset in repetition rate. The blue(red) shading highlights positive(negative) channels (*μ* = 0 denotes the pump laser) used in LiDAR experiments limited by the available amplifier bandwidth and amplified spontaneous emission noise. **d** Time-frequency map of heterodyne beat spectroscopy at the ±3 and ±11 channels. ENBW 2.45 MHz. **e** Time-frequency map of delayed homodyne beat spectroscopy. ENBW 2.45 MHz. Image peaks are related to imperfect phase compensation in IQ detection (cf. Supp. inf). **f** Channel-dependent frequency excursion bandwidth at the 100-kHz modulation frequency. **g** Channel-dependent absolute excursion difference of signal and LO combs. **h**, **i** Channel-dependent RMS deviation from a perfect triangular frequency chirp for LO and signal.

In the experiments, we used frequency combs with 99.39 GHz and 98.9 GHz repetition rates, i.e. a 490 MHz difference (cf. Fig. 2c). We simultaneously generated two DKS from a single triangularly chirped laser with an amplitude *B* = 1.5 GHz and a period *T* = 10 *μ*s and thermally tuned the two pump resonances into degeneracy to match their trajectories in the soliton existence range (cf. Fig. 2b). The pump laser frequency was modulated by dual Mach-Zehnder biased to single-sideband modulation. The voltage-controlled oscillator drove the modulator with a triangular waveform that we digitally predistorted and linearized (cf. Supplementary Information).

First, we obtained a heterodyne beatnote by superimposing the frequency combs individually with an external-cavity diode laser onto two balanced photoreceivers (BPD). The resulting signals were analyzed by short-time Fourier transform. Figure 2d shows the laser frequency of the two simultaneously chirped local oscillator and signal microcomb for channels *μ* = ±3, ±11, highlighting the similarity and relative spacing of the frequency modulation pattern. The delayed homodyne beat note spectrum for the same channels is depicted in Fig. 2e where the delay line distance corresponds to the frequency splitting.

The maximum number of LiDAR channels is limited by the optical amplification and coherent receiver bandwidths *B*_pd_. The latter limitation reads as *μ*Δ*f*_rep_ < *B*_pd_ and can be overcome by reducing the repetition rate difference Δ*f*_rep_ with the trade-off of a reduced distance ambiguity range. The channel-dependent frequency excursion *B*_*μ*_ (cf. Fig. 2f) is related to the soliton self-frequency shift induced by intrapulse Raman scattering^45,46^ and dispersive wave recoil^47,48^ and ranges from 1.3 GHz to 1.8 GHz, which corresponds to a native axial distance resolution Δ*x*_*μ*_ = *c*/2*B*_*μ*_ of 12 cm to 8 cm. The performance of dual-comb FMCW heterodyne detection relies not only on the mutual coherence of the Signal and LO combs (ensured by degenerate pumping scheme), but also on the equality (cf. Fig. 2g) and low nonlinearity (cf. Fig. 2h, i) of the chirp transduction from the pump laser in both nonlinear microresonators. The relative phase deviation between the corresponding Signal and LO comb lines (cf. Supplementary Figs. 4, 5) affects the resulting signal RF beatnote linewidth broadening and thus the LiDAR performance (outlined in the Supplementary Information).

### Massively parallel coherent ranging

**Fig. 3:**
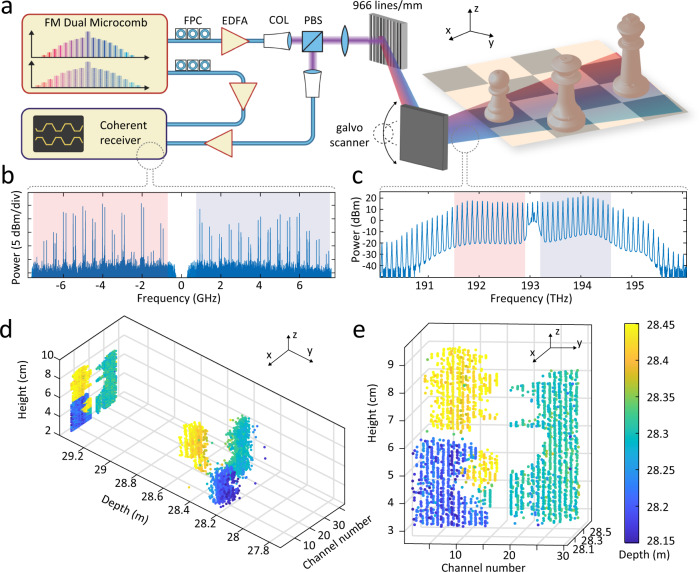
Dual-comb parallel 3D imaging. **a** Experimental setup. The amplified signal comb is dispersed in free space by a 966 lines/mm transmission grating, and a mirror galvanometer provides vertical scanning. EDFA Erbium-doped fiber amplifier, FPC Fiber polarization controller, COL collimator, PBS Polarizing beamsplitter. **b** Power spectral density of the electrical signal obtained at the coherent receiver highlighting 28 FMCW channels. The red and blue shading highlights signals obtained from negative and positive channel numbers. The resolution bandwidth equals to 100 kHz. Signal-to-Noise ratio ranges in between 5–20 dB with polarization-dependent variations. **c** Optical spectrum used for 3D imaging. The red and blue shaded regions correspond to the signal plotted in **b**. **d**, **e** Point clouds of the three chess figures obtained during a scan (28 × 136 points) of the mirror galvanometer.

To demonstrate the capabilities of dual-comb massively parallel coherent imaging, we perform proof-of-principle parallel ranging experiments. The setup is depicted in Fig. 3a. The soliton microcombs are amplified in erbium-doped fiber amplifiers (EDFA). The target comprises three chess figures (queen, king and pawn—cf. Supplementary Fig. 9) placed approximately ~1 m in front of the beam-splitter and optical transmission grating. A single-axis galvanometric mirror is used for beam scanning in the vertical direction. Figure 3c depicts the optical spectrum of the signal comb interrogating the target. The Fourier transform of the complex signal *I* + *iQ*, photodetected on the coherent receiver during 10 *μ*s, represents a two-sided spectrum containing information about all 28 channels (cf. Fig. 3b). Blue and red shadings highlight positive *μ* > 0 and negative *μ* < 0 frequency comb teeth with respect to the pump laser frequency. Two different projections of the 3D-imaging results for a scan of 136 vertical angles across the set of chess figures are depicted in Fig. 3d,e. A line of 28 pixels is recorded during a single 10 *μ*s triangular laser chirp, which equates to a true, i.e., bona fide 2.8 MPix/s coherent distance sampling rate at the sampling oscilloscope. We emphasize that this operation differs from experiments, which detect, digitize, and process each de-multiplexed channel successively and thus report aggregated data or sampling rates^22^. The details of the multichannel data segmentation filtering, IQ phase and amplitude imbalance compensation, computational complexity of the required signal processing, precision, accuracy and repeatability of distance measurements are outlined in the Supplementary Information.

### Dense megapixel per second coherent ranging

In a second proof-of-principle experiment, we demonstrate dense coherent hardware-efficient parallel velocimetry with our dual frequency-modulated soliton microcomb platform at even higher rates of 6.4 megapixel per second. Lowering the repetition rate to 35 GHz illustrates the advantage of the dual-comb approach, which alleviates the need to operate at large line spacings compatible with wavelength division multiplexers and facilitates mode spacing related limitations of channel isolation. To this end, we employ two low repetition rate soliton microcombs operating at *f*_rep_ of 35 GHz allowing us to have more than 60 channels within the Erbium-doped fiber amplifier (EDFA) gain window.

**Fig. 4:**
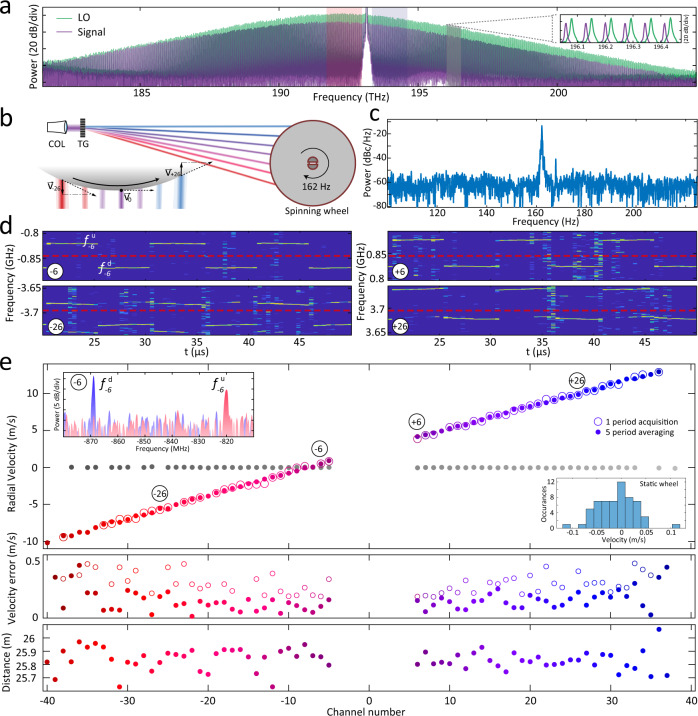
Dense dual-comb parallel velocimetry measurement at 6.4 megapixel/second rates. **a** Optical spectra of the 35 GHz dual FMCW combs. **b** Schematic illustration of the flywheel section irradiated by the signal comb lines. (Left) COL Collimator, TG transmission grating. (Right) The projections of the velocity *v*_*μ*_ of the wheel onto the comb lines. **c** Periodogram of the spinning flywheel sound recorded on a cellphone microphone depicting a peak corresponding to the rotation frequency. **d** Time-frequency map featuring an offset of the mean beat frequency from the *μ*Δ*f*_rep_ due to the Doppler shift. ENBW 2.45 MHz. **e** (Top) Multichannel velocity measurement for the flywheel rotating at 162 Hz for a single 10 *μ*s scan (open circles) and five frame stacking (filled circles). Grey data points show velocimetry results for the static wheel. The left inset shows the Fourier transform of the signal current in the RF band corresponding to *μ* = −6 channel recorded over one period. The right inset shows a static wheel velocity histogram. (Middle) Error of velocimetry for single scan and five frame stack. (Bottom) Ranging results for five frame stacking.

While spectral compression of the microcomb from 99 GHz to 35 GHz does limit the triangular chirp frequency excursion to 700 MHz at the same pump power, this does not limit the accuracy of Doppler velocimetry and we can retain the 100 kHz modulation frequency. The optical spectra of the signal and LO solitons are shown in Fig. 4a. The inset highlights the repetition rate offset Δ*f*_rep_ of ~140 MHz. The signal comb is dispersed by the same transmission grating along the circumference of a 20 mm flywheel rotating at 162 Hz (cf. Fig. 4b, c). The pump channel is approximately aligned at the center of the flywheel such that negative channels (negative RF frequencies) record an approaching target and positive channels (positive RF frequencies) a receding target. The time-frequency maps of the complex spectrum for the *μ* = ±6, ±26 channels are plotted in Fig. 4d, and the dashed red lines highlight the baseband frequencies of multiheterodyne detection equal to *μ*Δ*f*_rep_. We calculate the velocities by computing the mean deviation of the beat notes from the equivalent baseband and depict the results in Fig. 4e. Open circles correspond to the results after analyzing a single scan period, while the filled circles correspond to the averaging over five chirp periods. We also demonstrate a velocity profile of the static wheel (gray circles) for comparison. On average, we attained 56 pixel detections over one period resulting in 5.6 MPix/s, i.e., actually detected, velocity and distance information acquisition speed. We attribute the velocity measurement uncertainty (Fig. 4e middle panel) to the mechanical vibrations of the flywheel. The distance measurement is depicted in Fig. 4e bottom panel and is less accurate compared with ranging utilizing 100 GHz combs due to the reduced frequency excursion and, consequently, resolution and accuracy.

With recent demonstrations of DKS in low-repetition rate microresonators^49,50^ the approach could readily exceed 10 MPix/s.

## Discussion

In summary, we have demonstrated a megapixel-rate parallel coherent laser ranging based on multiheterodyne detection of chirped carriers on a single coherent receiver. Two integrated soliton microcombs driven by the same chirped pump laser provide a minimalist implementation of the dual chirped-comb system. The approach is free of channel separation, photo-detection and processing of individual channels.

Utilization of arbitrary, particularly very dense, frequency comb channel spacing is possible since multiplexers are not required. When combined with phased arrays, or other compact non-inertial scanning solutions, our approach provides a route to field-deployable MPix/s LiDAR systems that enable sufficient frame rate for video rate 3D imaging. Moreover, high-bandwidth silicon photonics-based IQ detectors are already offered commercially—making our method fully compatible with photonic integration. A recently demonstrated full heterogeneous integration combining InP/Si semiconductor lasers and ultralow-loss silicon nitride microresonators for DKS generation^51^ is feasible as a path to chip-scale parallel FMCW LiDAR. Such photonic integration does not only bring another degree of miniaturization and possibility of wafer-scale production but also reduces optical loss, increases noise performance of the laser, and achievable scanning rates^52^. Erbium-doped fiber amplifiers could be replaced by broadband semiconductor optical amplifiers (SOA) co-integrated on the silicon substrate^53,54^. It should be noted that SOAs are subject to high nonlinearities and spectral distortion. However, it can be reduced by selecting gain media with low *α*-factor^55,56^. The broadband amplification would increase comb repetition rate while maintaining high channel count, which leads to improved soliton comb line power and relaxes the requirements on the grating line density.

While the approach comes at the expense of reduced Signal-to-Noise ratio due to the multiheterodyne detection penalty^57^ (outlined in the Supplementary Information), it benefits from the absence of multiplexers or photonic integrated solutions for detection of individual channels, which typically exhibit significant insertion loss.

Synchronous tuning of the pump laser and the microresonator, i.e., using monolithically integrated piezoelectrical frequency tuners^58^ or frequency comb generation in electro-optical materials^20^, serves to eliminate the residual nonlinearities of tuning that arise from the Raman self-frequency shift of the soliton and remove the requirement of high-power pumping while possibly extending the soliton existence range.

Finally, and equally important, we believe that our work will motivate further investigation of the frequency swept microresonator dual-comb approach in the neighboring fields of linear and nonlinear spectroscopy, optical coherence tomography.

## Methods

### Sample fabrication

Integrated Si_3_N_4_ microresonators are fabricated with the photonic damascene process. Features are defined using deep-ultraviolet (DUV) stepper lithography, reactive ion etching and silica preform reflow prior to deposition reduces scattering losses. The waveguide width is 1.5 *μ*m, and its height is 0.82 *μ*m, which leads to an anomalous second-order dispersion of *D*_2_/2*π* = 1.13 MHz and the third-order dispersion parameter is *D*_3_/2*π* = 576 Hz. The positions of the resonance frequencies close to the pumped resonance are expressed with the series *ω*_*μ*_ = *ω*_0_ + ∑_*i*≥1_*D*_*i*_*μ*_*i*_/*i*!. The ring radius of the signal comb is 228.43 *μ*m and results in a resonator free-spectral range of *D*_1_/2*π* = 98.9 GHz. The LO comb has a similar cross-section, and its radius is 227.27 *μ*m, which leads to a free-spectral range of 99.35 GHz. Both resonators are operated in the strongly overcoupled regime with intrinsic loss rate *κ*_0_/2*π* = 15 MHz and bus waveguide coupling rate *κ*_ex_/2*π* = 130 MHz in order to optimize comb output power and optical signal-to-noise-ratio after amplification. The radii of the 35 GHz samples are 645 *μ*m and 648 *μ*m resulting in Δ*f*_rep_ ≈ 140 MHz.

## Supplementary information

Supplementary Information

Peer review file

## Data Availability

The data used to produce the plots within this paper are available at 10.5281/zenodo.5898523^59^.

## Code availability

The code used to produce the plots within this paper is available at 10.5281/zenodo.5898523^59^.
