# Supplementary material for: Dual chirped microcomb based parallel ranging at megapixel-line rates
Source: Nat Commun. 2022 Jun 7;13:3280. doi: 10.1038/s41467-022-30542-x (PMC9174235; doi:10.1038/s41467-022-30542-x)
Supplement: Supplementary file 1 — Supplementary Information [file 41467_2022_30542_MOESM1_ESM.pdf]

Supplementary information

Dual chirped microcomb based parallel ranging at megapixel-line rates

Anton Lukashchuk, Johann Riemensberger,* Maxim Karpov, Junqiu Liu, and Tobias J. Kippenberg†

Laboratory of Photonics and Quantum Measurements (LPQM),

Swiss Federal Institute of Technology Lausanne (EPFL), CH-1015 Lausanne, Switzerland

Dual FM soliton microcomb generation

We use an external cavity diode laser (ECDL) for our proof-of-principle, which is coupled to a dual Mach-Zehnder Modulator driven by a frequency-agile VCO and biased for single-sideband modulation. The laser is amplified, split and coupled into two photonic chips using lensed fibers and double inverse tapers [1]. Optical power incident on the each chip is ∼2 W. Manual tuning with the ECDL piezo is used to tune into resonance. Upon traversing from blue to red, the chaotic modulation instability state collapses into a stable dissipative Kerr soliton [2] state. The thermal nonlinearity of Si_3N_4 facilities the elimination of undesired multi-soliton states [3] when the laser-cavity detuning $\delta^{\mathrm{sig,LO}}$ is reduced beyond the soliton existence range into the transient chaos region [4]. If both cavity resonances are aligned, the associated sudden drop in intracavity power increases the detuning δ of the switching comb, hence leapfrogging the detuning of the non-switching comb. The laser detunings are monitored using the phase modulation response technique introduced in [3] and temperatures of the samples are adjusted during the dual soliton switching process, if necessary using a thermal tuner. In this way, dual single soliton states can be obtained routinely and quickly. We subsequently optimize the laser-cavity detunings $\delta^{\mathrm{sig,LO}}$ of signal and LO combs by thermal tuning to minimize the differential Raman shift and hence optimize linearity.

* johann.riemensberger@epfl.ch

† tobias.kippenberg@epfl.ch

Linearization

The precision of coherent laser ranging is directly impacted by the linearity of the chirps that constitute the triangular frequency modulation. The chirp is applied to narrowband CW laser via single side-band modulator driven by a VCO. An iterative linearization algorithm was applied to the pump frequency chirp based on chirp measurement in an imbalanced Mach-Zehnder interferometer (MZI) [5]. The detailed procedure can be found in the Methods section of Ref. [6]. We estimate the root mean square nonlinearity (the deviation of the instantaneous frequency from the perfect triangular trace) of the FMCW comb sidebands to be below 5 MHz for sweeping rate of 100 kHz [6]. The 3dB modulation frequency cut-off that frequency combs are able to reproduce appears to be at least 40 MHz. The maximum tuning rate, i.e. the product of excursion and tuning frequency is determined by the effective filter cavity response time (photon decay rate) and should follow $d\Delta/dt < (\kappa/2\pi)^2$, where κ is a loaded cavity linewidth and equals ≈ 150 MHz in our case.

Parallel velocimetry and ranging

The experimental setup is illustrated in Supplementary Fig. 7a. The frequency modulation $1/T$ and excursion B of the pump laser are adjusted to 100 kHz and 1.55 GHz, respectively. A pump laser with triangular frequency modulation drives two distinct optical microresonators with slightly different radii, which serve as signal and LO in the experiment. After filtering (FBG) and amplification (EDFA), the signal comb is spatially dispersed over the target area using diffractive optics (transmission grating (966 lines/mm)). Each signal comb tooth μ represents an independent FMCW ranging channel measuring distance x_μ and velocity v_μ. The reflected signal is post-amplified similar to signals in high-bandwidth and long haul optical telecommunication systems and simultaneously superimposed with the amplified LO comb on a coherent receiver. A programmable filter (WS) is used to filter out excess (amplified spontaneous emission) ASE noise around the pump of the LO. A bistatic detection with separate collimators for the transmit and receive path is chosen to minimize spurious backreflection in the fiber components. Another balanced photodiode is used to calibrate Δf_{rep} and the channel-dependent frequency excursion B. A mirror galvanometer (shown in Fig. 3) is used to scan the dispersed light beams in the vertical direction.

The imaging experiment (Fig. 3) is carried out with a signal soliton microcomb repetition rate of 98.9 GHz and repetition rate difference of 490 MHz. The in-phase (I) and quadrature

(Q) signals from the coherent photoreceiver are recorded on a fast oscilloscope and processed offline. We utilize microcomb channels between $\mu = -15, +15$, where $\mu = 0$ denotes the pump frequency, while $\mu = 0, \pm 1$ are filtered out by the WS due to the excess of the ASE, resulting in 28 independent channels. The required coherent receiver bandwidth for this configuration is determined as $\mu \Delta f_{\mathrm{rep}} \approx 7.5$ GHz. All the data points are collected from a single trace covering 136 vertical lines. The galvo-scanner is set on a linear scan mode, while the oscilloscope is triggered in segmential acquisition mode with 10 μs segment scan time and 10 ms idle time between segments to allow the scanning Galvanometer enough time to rotate and capture the full scene. Overall, the measurement time is less than 1.5 seconds and was limited by the galvanometer response bandwidth, not the pixel sampling rate. The 3D point cloud of 136×28 pixels was obtained after post-processing. The test scene is presented in Supplementary Fig. 9. Chess figures are placed approximately one meter after the transmission grating, the differential delay between signal and LO combs in optical fiber is around 27 m. The Pawn and Queen figurines are spaced 10.5 cm apart while the Queen and King figurines are spaced 13.5 cm apart. The relative distance accuracy is depicted on Supplementary Fig. 10 by plotting histograms of detected pixels for both positive and negative channels. To estimate the overall distance precision of the dual comb LiDAR we perform ranging experiment of a static object. Channel-dependent precision is obtained by measuring the distance corresponding to each individual channel during 49 chirp periods (490 μs) and calculating the standard deviation (Supplementary Fig. 11). Precision of our system ranges from 1 to 5.5 cm. The precision value is governed by how precisely one can define the RF frequency beatnote. It depends both on the frequency bin spacing determined by the target interrogation time and nonlinearities in the system that broaden the beatnote. In our system, Raman nonlinearity degrades the comb line precision towards higher $|\mu|$ since its impact is proportional to the relative comb number (described below).

The velocimetry measurements are carried out with 35 GHz microcombs and 140 MHz Δf_{rep} allowing us to increase the number of operational channels $\mu = \pm 5, \pm 40$. Channels $\mu = -4, +4$ are filtered out by the WS due to the excess ASE. The frequency excursion B_μ ranges from 500 up to 950 MHz (Supplementary Fig. 7d), which results in decreased depth resolution compared to the previous 100 GHz soliton microcomb system. The flywheel is rotating at frequency 162 Hz resulting in the 20.4 m/s tangential velocity.The velocity errors depicted in Fig. 4e are attributed to the mechanical vibrations of the flywheel. The velocity error of a single measurement is defined by calculating a variance of the Gaussian fitted to the beatnote. For five consecutive measurements a standard deviation of $N \leq 5$ detected

velocity values is calculated for every channel. Mechanical vibrations of the flywheel not only impact distance and velocity precision, but also limit the total number of possible detections. In Supplementary Fig. 12, we present the detection statistics obtained over 190 μs continuous measurement time, i.e. 19 periods of the triangular waveform. Unfilled circles in panel a) (the same as in Fig. 4d) correspond to a measurement over one period with a mean number of detections equal to 56. Filled circles correspond to the data averaged over five periods. Panel b) depicts a probability for each channel to be detected. The roll-off at high $|\mu|$ originates from the limited optical amplification bandwidth. Panel c) represents the probability distribution of the sum of successfully detected pixels during individual scan periods, which is calculated by dividing the number of detections in a period over the full number of channels ($\mu = -40, +40$).

Coherent detection and post-processing

The frequency of the complex heterodyne beat note for the channel μ and a photon time-of-flight τ follows as the difference between the instantaneous optical frequencies of signal and LO comb teeth $\nu_\mu^{\mathrm{sig,LO}}$

$$\begin{aligned} f_\mu^{\mathrm{IQ}}(t) &= \nu_\mu^{\mathrm{LO}} - \nu_\mu^{\mathrm{sig}} \\ &= \delta(t) + \mu f_{\mathrm{rep}}^{\mathrm{LO}}(t) - \delta(t+\tau) - \mu f_{\mathrm{rep}}^{\mathrm{sig}}(t+\tau). \end{aligned} \tag{1}$$

The first and third terms are similar to the case of single frequency coherent photoreceiver FMCW LiDAR [7]. In our case that frequency is offset by the repetition rate difference of the soliton microcombs multiplied by the channel number and adding the Doppler shift due to the relative target velocity v, we arrive at the following expressions:

$$\begin{aligned} f_\mu^{\mathrm{u}} &= \mu \cdot \left(f_{\mathrm{rep}}^{sig} - f_{\mathrm{rep}}^{LO}\right) + \frac{B}{2T} \cdot \tau + \nu_\mu \cdot \frac{v}{c} \\ f_\mu^{\mathrm{d}} &= \mu \cdot \left(f_{\mathrm{rep}}^{sig} - f_{\mathrm{rep}}^{LO}\right) - \frac{B}{2T} \cdot \tau + \nu_\mu \cdot \frac{v}{c}. \end{aligned} \tag{2}$$

We point out that the linewidth of beatnote f_μ^{IQ} depends on the relative phase deviations between the Signal and LO comb lines (Supplementary Figures 4,5) that inherent the chirp from single FMCW pump laser. This places additional requirements on linearity and uniformity of chirp transduction during DKS generation compared to the single comb case (Supplementary Figures 2,3).

According to our notation, positive frequencies in the complex RF spectrum correspond to optical carrier frequencies larger than than the pump, negative frequencies to channels

with smaller optical carrier frequencies than the pump. The distance and velocity can be inferred from these expressions and are depicted in the inset of Fig. 1d.

Our coherent receiver consists of 90° optical hybrid coupler, two balanced photodetectors and subsequent RF amplifiers. This type of receiver is commonly referred as "phase-diversity homodyne receiver" [8, 9] or "intradyne receiver" [10]. It is similar to established receivers used in quadrature-amplitude modulation schemes that are employed in long haul optical communication systems. It allows full reconstruction of the amplitude and phase of the RF beat note between the signal and the LO and reveals distinct spectral information both in positive and negative RF frequencies. Due to the differential delays of discrete components and the response of the balanced photoreceivers and amplifiers, the in-phase (I) and quadrature (Q) signals are not perfectly orthogonal. We perform IQ imbalance correction [11], which substantially improves orthogonality (Supplementary Fig. 6). However, for higher channels mismatch still exists and one can observe 'images' on time frequency maps (i.e. Fig 2e left panel).

The post-processing relies on short time Fourier transform over the half of the period to retrieve $f_\mu^{\mathrm{u}}, f_\mu^{\mathrm{d}}$ RF frequencies. In this regard, the 'image' peaks do not pose a problem, since we know that the positive frequencies will give a higher frequency beat note first while for the negative ones it would be the lower one (Supplementary Fig. 6). It is obtained by triggering data acquisition on the oscilloscope by function generator used to create the triangular frequency modulation control signal for the VCO. Distance information is obtained as a difference between $f_\mu^{\mathrm{u}}, f_\mu^{\mathrm{d}}$, while the mean offset of $f_\mu^{\mathrm{u}} + f_\mu^{\mathrm{d}}$ from $\mu\Delta f_{\mathrm{rep}}$ is proportional to the velocity. Calculation of the Δf_{rep} is outlined below. Further improvements, especially in long range detection can be achieved using active demodulation analysis [12].

To evaluate the real-time digital processing requirements, we give an estimate of computational complexity. The main computational operation is a discrete Fourier transform (DFT), which can be computed via the Fast Fourier transform (FFT) method. The complexity of FFT can be estimated as $\approx 4\mathrm{N}^*\log_2(\mathrm{N})$, where N is a sample size [13]. Considering a coherent receiver of 10 GHz bandwidth detecting 40 comb lines (channels) with an FM period of 10 μs, one requires 2 DFTs for the up-ramp and down-ramp of the chirp. Let the sampling speed be 20 GS/s fulfilling the Nyquist requirement. The number of the sampled points for one ramp (half of the period) is 20 GS/s * 5μs = 10^5. The required number of operations for FFT is $4\mathrm{N}^*\log_2(\mathrm{N}) = 6.64^*10^6$. Next we use Gaussian fitting for peak detection in every particular frequency band. For nonlinear fitting, we take 20 points covering 20 / 5μs = 20*200 kHz = 4 MHz band, which is broader than the beatnote linewidth.

These operations require a peak search and threshold detection up to 40 times in intervals of $10^5/40$ points (O(N) - complexity) and to fit a Gaussian for 20 points surrounding every maximum ($O(\mathrm{N}^3)$ - complexity). The complexity of the latter 2 operations is more than an order of magnitude lower than the number of operations needed for FFT. Thus we estimate the computational requirement to be ≈1.33 TFlops rate. A commercial FPGA Artix-7 by Xilinx (price ≈100$) could handle this task with 930 GMACs (≈1.86 TFlops).

Calibration of channel-dependent frequency excursion

In general, the soliton repetition rate f_{rep} depends on the laser cavity detuning, because of intrapulse stimulated Raman scattering [14, 15] and the soliton recoil effect associated with dispersive wave emission[16, 17]. During chirped soliton generation, this induces a change of the soliton repetition rate during each chirp cycle, which is observed in the form of a channel-dependent frequency excursion B_μ and chirp nonlinearity [6]. We observe that it does not depend strongly on the pump laser detuning and is independent of the pump power.

We measure the time-dependent chirp on both signal and LO combs and all channels by recording a heterodyne beat note with a second laser simultaneously on a pair of balanced photodetectors and a fast sampling oscilloscope (cf. Fig. 2a). For direct comparison of signal and LO chirps, we add the two beat note signals prior to short-time Fourier transform and depict both beat notes on a single panel. The full dataset corresponding to the subset of heterodyne beat signals presented in Fig. 2d is depicted in Supplementary Figure 1. The amplitude of the triangular ramps decreases from positive to negative channels both for LO and signal. We retrieve the frequency excursion by fitting a symmetric triangular ramp to the time-dependent frequency of the heterodyne beat notes. The result of this analysis is plotted in Fig. 2f-i.

Heterodyne beat spectroscopy is well suited to characterize the chirp waveforms, but not practical for ranging and velocimetry, as it requires an independent reference laser. Hence, during the experiments presented in figures 3 and 4, we utilize a reference optical fiber MZI that is derived by tapping a fraction of signal and LO and beating them together on a second balanced photodiode (cf. Supplementary Fig. 7a). Inphase detection suffices, because Doppler-shifts are negligible in the reference MZI and all channels μ observe the same distance x_μ but different frequency excursions B_μ, which simplifies interpretation of the signal, which is plotted in Supplementary Figure 7b.

Due to the channel-dependent excursion $\pm\mu$ channel would give a beat note consisting

of four lines (cf Supplementary Fig. 7b), where two outer lines correspond to positive frequencies with higher excursion and two inner lines correspond to negative frequencies with lower excursion. Thus, we perform excursion inference for the 6 channels $\mu = \pm 1, \pm 3$ (that is allowed by 1.6 GHz bandwidth) and further extrapolate it considering linear dependence (cf. Fig. 2 f,h,i). For the 35 GHz chips we utilize channels $\mu = \pm 2, \pm 10$ for calibration. Knowledge of the pump excursion enables us to calculate the distance of MZI, while given distance one can obtain excursion for channel μ from time-frequency analyzes. The same way one can infer channel-dependent excursion from real distance measurement if the distance to the target is the same for all of the channels (e.g. carton block). Furthermore, we can also calculate Δf_{rep} from the same calibration measurement. Since there is no Doppler shift, Δf_{rep} is equal to the mean of two beat notes divided by $|\mu|$. Hence, we are able to conduct the experiments with free-running soliton microcombs. Supplementary Fig. 7c,d present comparison of the excursion inferred from the calibration measurement and further extrapolated and from a real distance measurement with a carton block. In the experiments described in the main part of the manuscript we utilized excursion and Δf_{rep} for velocity and distance calculations inferred from the calibration measurements.

Impact of nonlinear chirp transduction

Multiheterodyne mixing of dual-chirped soliton microcombs necessitates not only soliton generation to preserve chirp linearity from the pump to the comb teeth, but also requires that chirps are transduced equally, so as to avoid a difference in the frequency excursion B_μ between the signal and LO comb teeth that would broaden the ranging beatnote and penalize detection precision and sensitivity. We have two contributions effecting soliton repetetion rate given change in pump frequency: the chromatic dispersion of the cavity D_2, i.e. frequency dependency of the free-spectral-range D_1, and Raman shift Ω [14, 15]. The overall change in repetition rate as a function of the detuning δ (frequency difference between the pump and 'cold' cavity resonance) can be written as

$$f_{\text{rep}}(\delta) = f_{\text{rep}}(0) + (\delta + \Omega(\delta))\frac{D_2}{D_1}. \tag{3}$$

For dielectric integrated microresonators it is generally found that $\delta \ll \Omega(\delta)$ and hence the first term in the round brackets can be omitted. We also neglect the weak dependence of the free-spectral range and dispersion on the laser-cavity detuning that can be derived as $D_n(\delta) - D_n(0) \approx \delta \cdot D_{n+1}/D_1 \ll D_n(0)$. The linear dependence of the instantaneous f_{rep}

on the laser-cavity detuning δ induces a channel-dependent frequency excursion B_μ without introducing nonlinearity into the triangular chirp [6]. Non-linear coupling bestween f_{rep} and f_{ceo} during the detuning sweep due to the soliton self-frequency shift leads to distortions of the triangular chirp. The nonlinear relation between the laser cavity detuning and the Raman soliton shift was derived in Ref. [15]:

$$\delta = \sqrt{\frac{15c\beta_2\omega_0}{32nQ}\frac{\Omega}{\tau_R}} - \frac{c\beta_2}{2n}\Omega^2, \tag{4}$$

where τ_R is Raman shock time and $\beta_2 = -\frac{n}{c}D_2/D_1^2$ is the chromatic dispersion term. Supplementary Fig. 8 a,b depict the time-varying detuning $\delta(t)$ of a triangular chirp sequence of the pump laser and the Raman induced soliton self-frequency shift $\Omega/2\pi$ caused by the variation of the detuning. Supplementary Fig. 8 c depicts the induced variation of f_{rep} (blue) and Δf_{rep} component (red). We investigate the degradation of chirp linearity and similarity between the signal and LO combs due to the Raman effect as function of their respective and in general dissimilar detunings $\delta^{\mathrm{sig,LO}}$ by inserting the Raman induced periodic change of f_{rep} into equations 1 and 2. We distinguish the cases of vanishing and modest differential detunings $\Delta\delta = \delta^{\mathrm{sig}} - \delta^{\mathrm{LO}} = 10MHz$ (cf. Fig. 8 d,e,f).

The small difference in f_{rep} between signal and LO microcombs results in different Raman shifts, which causes frequency excursions to differ between the signal and LO combs on the scale of 1 MHz, which is comparable to the induced chirp nonlinearities of pump to side-band chirp transduction (Supplementary Fig. 8 c,e,f). According to the equations 2 Raman nonlinearity scales with the relative mode number from the pump. This can be directly seen from Supplementary Figures 4,5, where relative phase deviation between corresponding Signal and LO comb lines and their phase noise power spectral densities (PSD) $S_{\phi\phi}(f)$ increase for higher $|\mu|$. Values in the top right corners of Supplementary Figure 5 denote integral of the PSD . A value less than $2/\pi$ heuristically corresponds to the Fourier transform limited linewidth, since $\int_{\frac{\Delta\nu}{2}}^{\infty} S_{\phi\phi}(f)\, df = 2/\pi$ [18]. Increased linewidth of the detected beatnotes results in the reduced resolution and degraded SNR, as well as impaired distance precision (Supplementary Figure 11). Substantially higher phase deviations in channels $+11, +3$ are attributed to the presence of mode crossings in the microresonator spectra [17]. Similarly, phase deviations in channel -1 do originate from unfiltered ASE noise. To determine the distance inference degradation due to the soliton induced Raman self-frequency shift, we simulate the full signal generation and coherent detection chain at a distance of 30 m with a custom `MATLAB` script and apply the same data analysis techniques to the artificial (i.e. numerically generated) multiheterodyne LiDAR trace as for the real data. The resulting

time frequency traces are depicted in Supplementary Figure 8 g,h,i for the pump and the 15th low and high frequency comb teeth. The overall manifestation of the soliton Raman self-frequency shift induced nonlinearity can be seen in the tilt and curvature of the time dependent complex RF beat note frequency. The effects of the nonlinearities are exacerbated for channels further away from the pump.The curvature that is imposed by the nonlinear component of the Raman frequency shift leads to a bias of the measured distances that increases with length difference between the target and the calibration MZI, differential detuning $\Delta\delta$ and channel number (cf. Supplementary Figure 8 j,k,l,m). In the ranging experiments presented here, we calibrate the bias by measuring the static target with the same distance for all the channels and apply this correction for further ranging experiments.

In a real-world deployment of our system, a feed-forward scan scheme [19] or phase-locking the comb to a tuned resonator [20] to avoid detuning-dependent changes of the microcomb repetition rates would be favorable, and are possible to avoid this effect. Co-integration of both microcomb resonators on a single chip to avoid differential frequency drift is equally straightforward.

Signal-to-noise ratio of multiheterodyne FMCW LiDAR

A significant advantage of heterodyne detection is that it can approach the photon shot noise limit of detection and attain single photon sensitivities for low signal powers on a conventional semiconductor photodiode, if sufficient LO power is supplied for amplification. In the multiheterodyne case all individual channel LO are impinging on a small number of photodiodes (4 in case of a phase diversity receiver with balanced photodiodes). Generally, for a heterodyne LiDAR detection $P^{\text{LO}} > P^{\text{sig}}$ and the shot noise (mainly contributed by the LO) is a dominating source of noise (i.e., higher than the thermal noise) if sufficient LO power is supplied to the photodiode.

$$\text{SNR} = \frac{\langle I^2 \rangle}{\langle \Delta I^2 \rangle} = \frac{P^{\text{sig}}}{\hbar\omega B_{\text{RF}}} \tag{5}$$

Where B_{RF} is the resolution bandwidth of the analysis FFT. For simplicity, we consider quantum efficiency of the photodiode to be 100%, thus the electric current is $I = RP = \frac{e}{\hbar\omega}P$. If an optical amplifier is used, than for the shot noise limited detection the total SNR would be at least 3dB lower:

$$\text{SNR} = \frac{P^{\text{sig}}}{2\hbar\omega B_{\text{RF}}} \tag{6}$$

Below we elaborate a more detailed analyzes for the case of multiple channels detected on a single balanced photodiode. Consider N distinct signal-LO channels distributed over the photodiode bandwidth with equal powers of the signal channels $P_\mu^{\mathrm{sig}} = P^{\mathrm{sig}}$ and LO channels $P_\mu^{\mathrm{LO}} = P^{\mathrm{LO}}$ for $\mu \in [1, N]$. For heterodyne detection the photodiode current of channel μ is $2R^2 P^{\mathrm{LO}} P^{\mathrm{sig}}$, while the total noise $\langle \Delta I^2 \rangle$ effecting channel μ would consist of shot noise, thermal noise, spontaneous-spontaneous beating noise, and signal/LO-spontaneous beating noise [21].

The photon shot noise is proportional to the mean current impinging on the photodiodes

$$\begin{aligned} \sigma_{\mathrm{sh}}^2 &= 2q\langle I \rangle B_{\mathrm{RF}} \\ &= 2qR(NGP^{\mathrm{LO}} + NGP^{\mathrm{sig}} + P_{\mathrm{ase}}^{\mathrm{LO}} + P_{\mathrm{ase}}^{\mathrm{sig}})B_{\mathrm{RF}} \\ &\approx 2qRNGP^{\mathrm{LO}} B_{\mathrm{RF}} \end{aligned} \tag{7}$$

mainly contributed by N LOs. Where P_{ase} is spontaneous emission noise and it equals $\rho_{\mathrm{ase}} \Delta\nu_{\mathrm{amp}} = n_{\mathrm{sp}} \hbar\omega (G-1) \Delta\nu_{\mathrm{amp}}$ with the amplification bandwidth $\Delta\nu_{\mathrm{amp}}$ (4 THz for EDFA) and the spontaneous emission factor $n_{\mathrm{sp}} = 1$ for the amplifier with complete inversion, q is the electron charge. Additionally, we consider equal gains for the detector pre-amplifiers of the signal and local oscillator microcombs $G^{\mathrm{LO}} = G^{\mathrm{sig}} = G$.

We consider the thermal noise of the photodiodes at room temperature

$$\sigma_{\mathrm{th}}^2 = 4\frac{kT}{Z} B_{\mathrm{RF}} \tag{8}$$

where kT is a thermal energy and the impedance Z of the load is 50 Ohm. Spontaneous-spontaneous and signal/LO-spontaneous beating noises include cross terms only, because we employ balanced photodetection,

$$\sigma_{\mathrm{sp-sp}}^2 = 4R^2 \rho_{\mathrm{ase}}^{\mathrm{sig}} \rho_{\mathrm{ase}}^{\mathrm{LO}} \Delta\nu_{\mathrm{amp}} B_{\mathrm{RF}}, \tag{9}$$

$$\sigma_{\mathrm{sig/LO-sp}}^2 = 4R^2 (NGP^{\mathrm{LO}}) \rho_{\mathrm{ase}}^{\mathrm{sig}} B_{\mathrm{RF}} \tag{10}$$

$$+ 4R^2 (NGP^{\mathrm{sig}}) \rho_{\mathrm{ase}}^{\mathrm{LO}} B_{\mathrm{RF}}. \tag{11}$$

In the experiment, we use two optical amplifiers for the signal comb: a booster-amplifier before free-space emission and a detection pre-amplifier after light collection. In the above equations, P^{sig} denotes the optical power of the signal comb line at the input of the 90° optical hybrid. The ASE noise of the booster amplification stage is irrelevant in the coherent ranging application, as the optical loss in free space (> 60 dB) generally surpasses the single stage amplification gain. Thus the photon shot noise would be dominating at the

post-amplification stage. Combining all noise terms and neglecting minor contributions, we determine the SNR for a given channel:

$$\mathrm{SNR}_\mu = \frac{\langle I_\mu^2 \rangle}{\sigma_{\mathrm{sh}}^2 + \sigma_{\mathrm{th}}^2 + \sigma_{\mathrm{sp-sp}}^2 + \sigma_{\mathrm{sig/LO-sp}}^2} \tag{12}$$

$$\approx \frac{2R^2 P^{\mathrm{sig}} G P^{\mathrm{LO}}}{4R^2 (NGP^{\mathrm{LO}}) \rho_{\mathrm{ase}}^{\mathrm{sig}} B_{\mathrm{RF}}} \approx \frac{P^{\mathrm{sig}}}{2N\hbar\omega B_{\mathrm{RF}}} \tag{13}$$

Thus, in the case of shot-noise limited operation, the SNR of a channel μ is reduced by the shot noise of the additional local oscillators and N times lower than in case of its detection on N separate photodetectors. This multiheterodyne penalty is well known in the realm of dual comb spectroscopy [22] and amounts to 17 dB for 50 channels. It can be reduced by spectral slicing or interleaving. While the approach comes at the expense of reduced SNR due to the multiheterodyne detection penalty, the latter is compensated for by the absence of multiplexers or photonic integrated solutions for detection of individual channels, which typically exhibit significant insertion loss.

SUPPLEMENTARY FIGURES

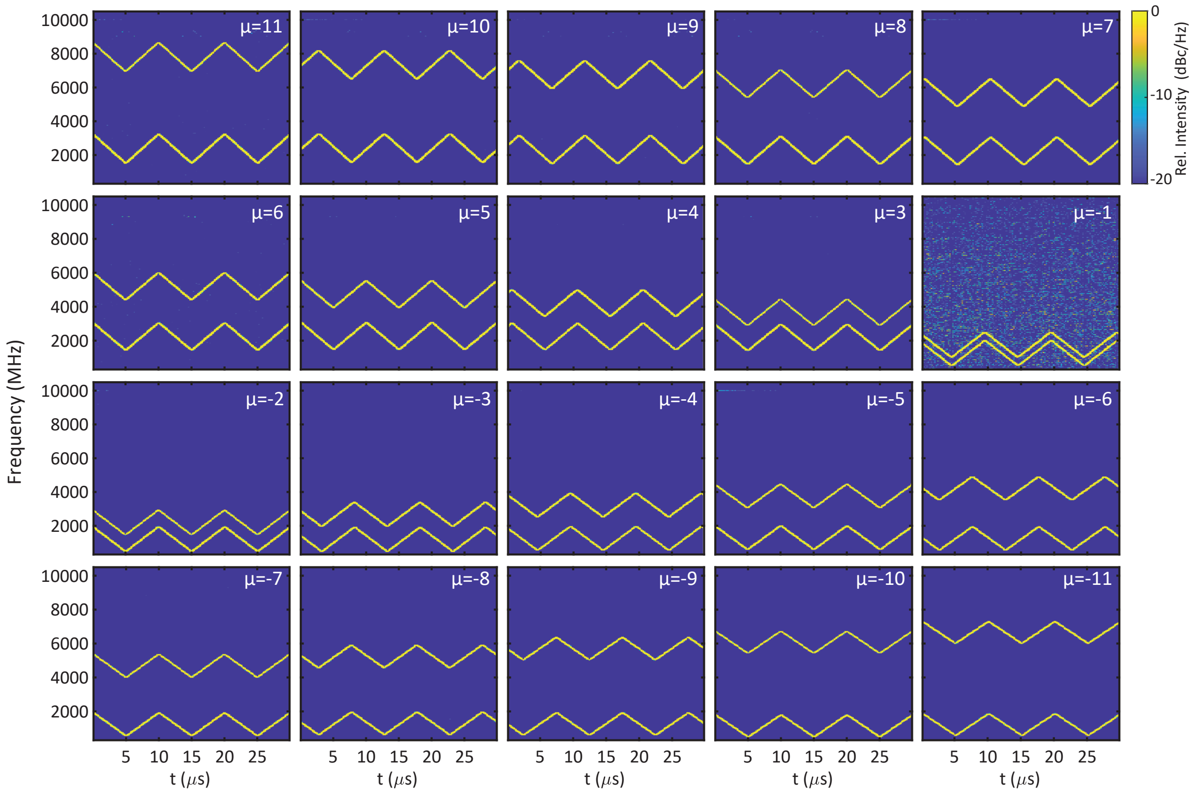

Supplementary Fig. 1. **Heterodyne measurement of FMCW Dual-Comb.** Time-frequency map of heterodyne beat spectroscopy of signal and LO microcombs with external reference obtained by short-time Fourier transform with resolution bandwidth 2.45 MHz. Depicted here is the full data set corresponding to Fig. 2d of the main manuscript.

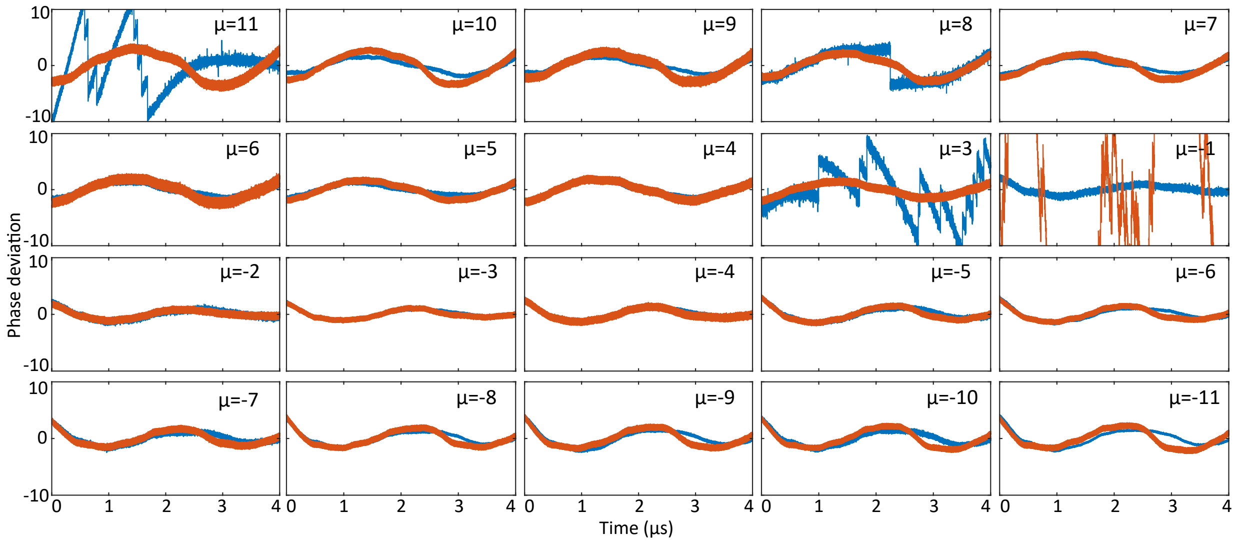

Supplementary Fig. 2. **Phase deviation of the chirped comb lines.** Blue/Red - Signal/LO comb line. The phase of the signals was obtained via the Hilbert transform. The subsequent subtraction of quadratic fit (corresponds to linearly changing frequency of the chirp) results in phase deviation.

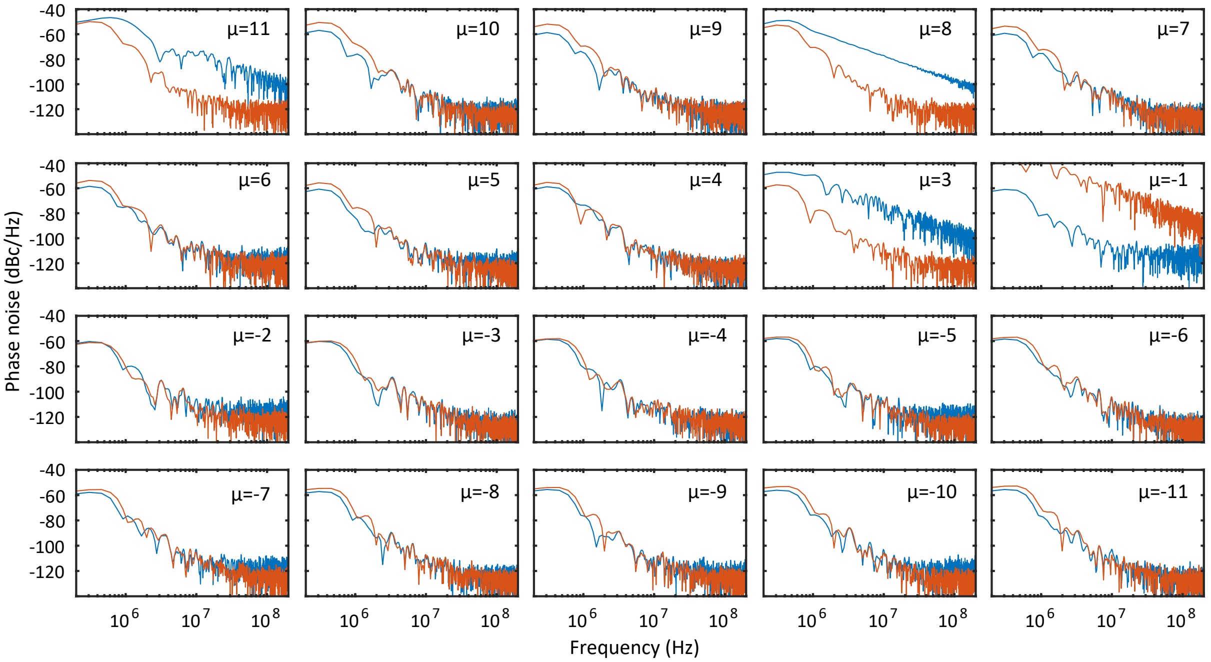

Supplementary Fig. 3. **Phase noise of Signal and LO combs.** Blue/Red - Signal/LO comb line.

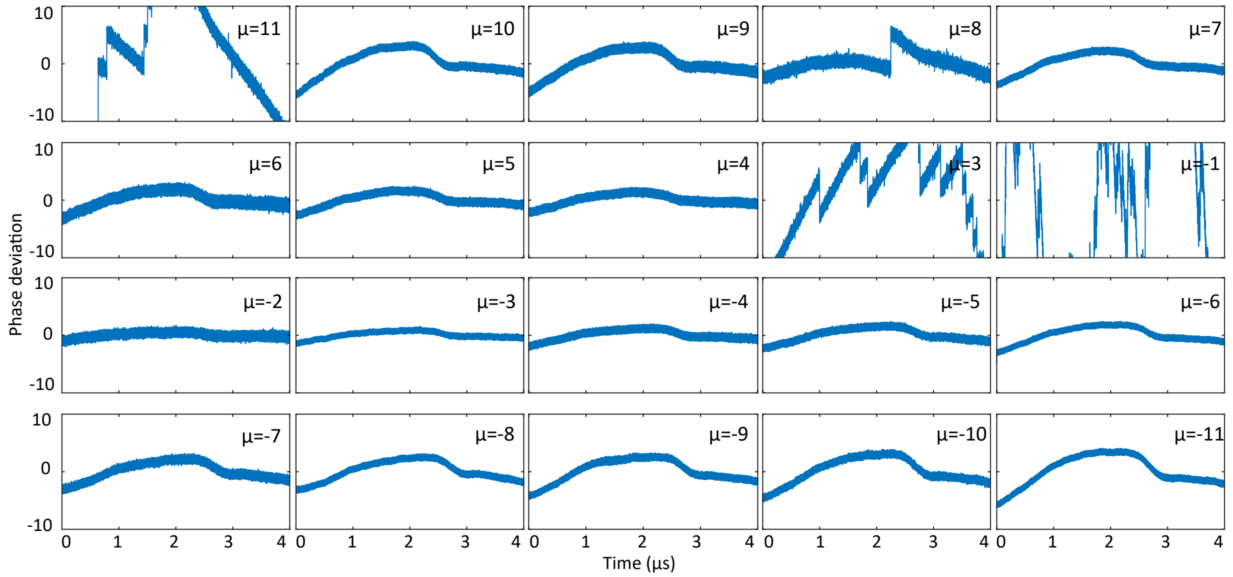

Supplementary Fig. 4. Differential phase deviation of the chirped comb lines. The phase deviation is obtained by subtracting the phase of the Signal comb from the phase of the LO comb for a given channel and subsequent subtraction of linear fit.

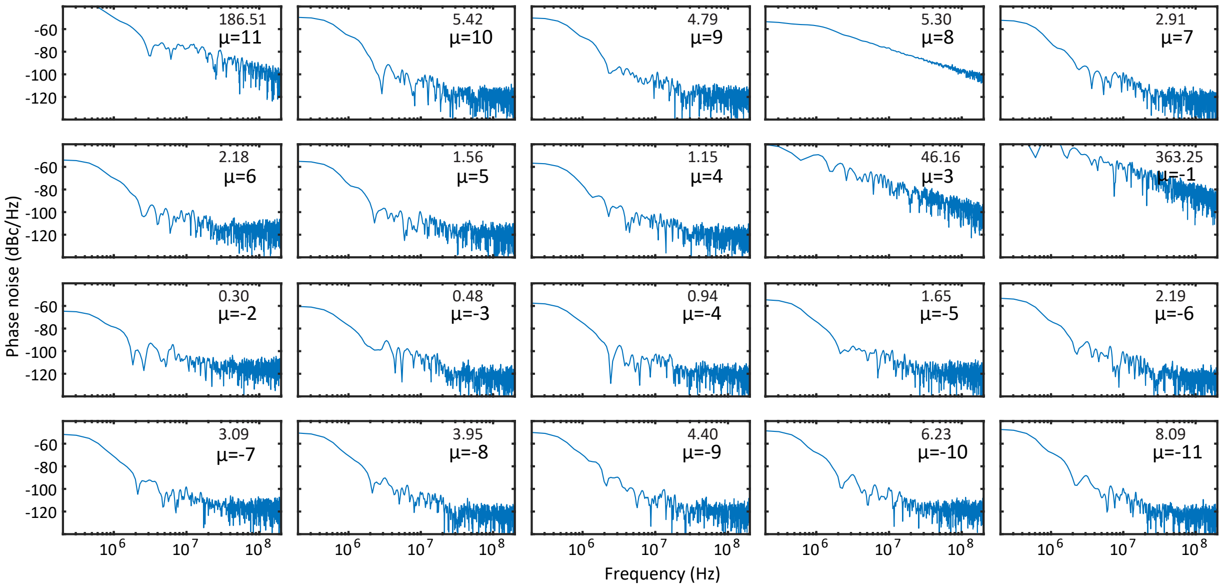

Supplementary Fig. 5. **Phase noise of the beatnote signal between Signal and LO.** The number in the top right of every plot corresponds to the integral of the PSD. Heuristically, the value less than $2/\pi$ corresponds to the Fourier-limited beatnote linewidth.

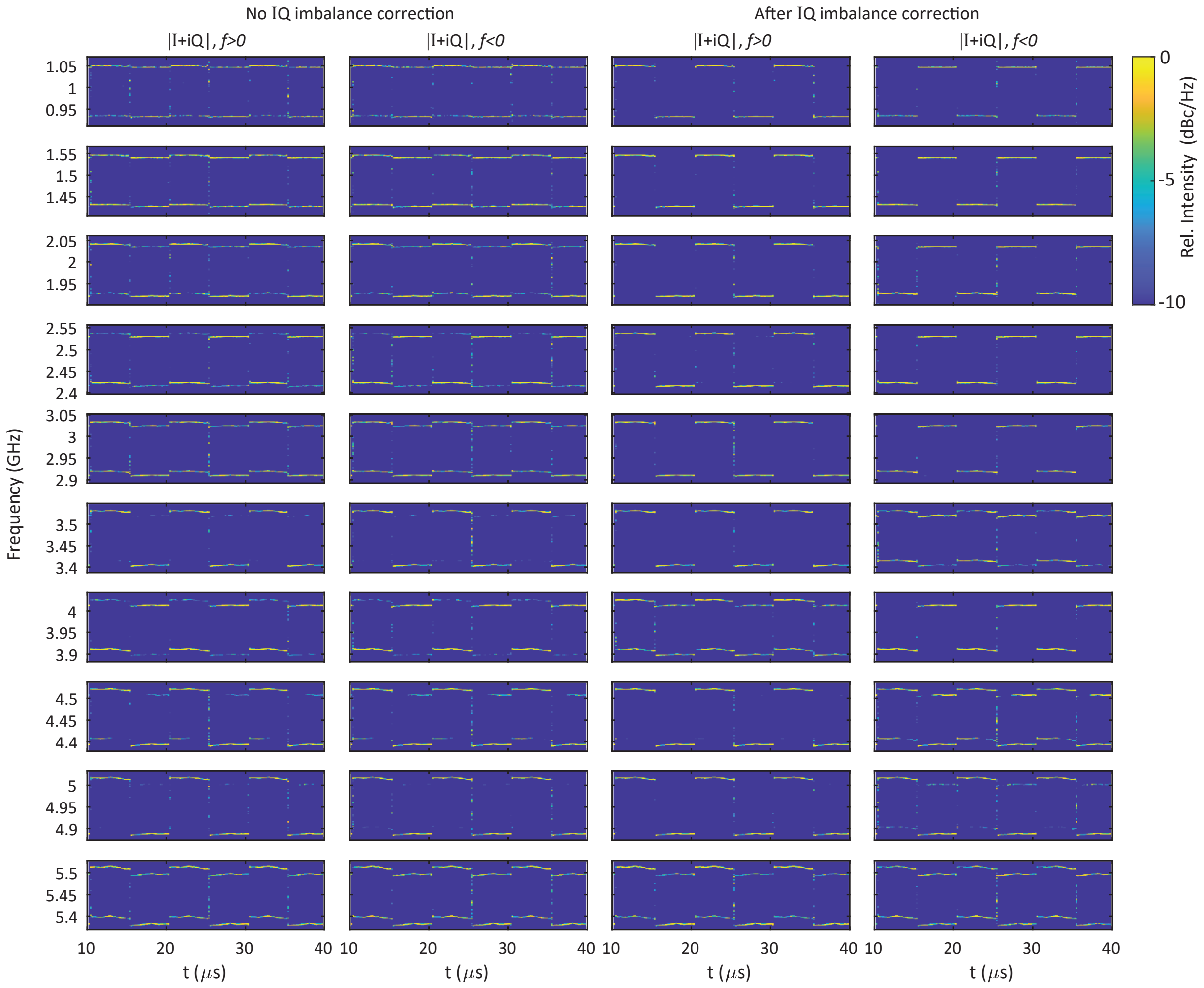

Supplementary Fig. 6. **IQ imbalance correction.** Time-frequency map of delayed heterodyne beat spectroscopy for $\mu = \pm 2, \pm 11$. First two columns correspond to positive and negative channels without IQ imbalance correction, while second two columns are with IQ imbalance correction. ENBW 2.45 MHz.

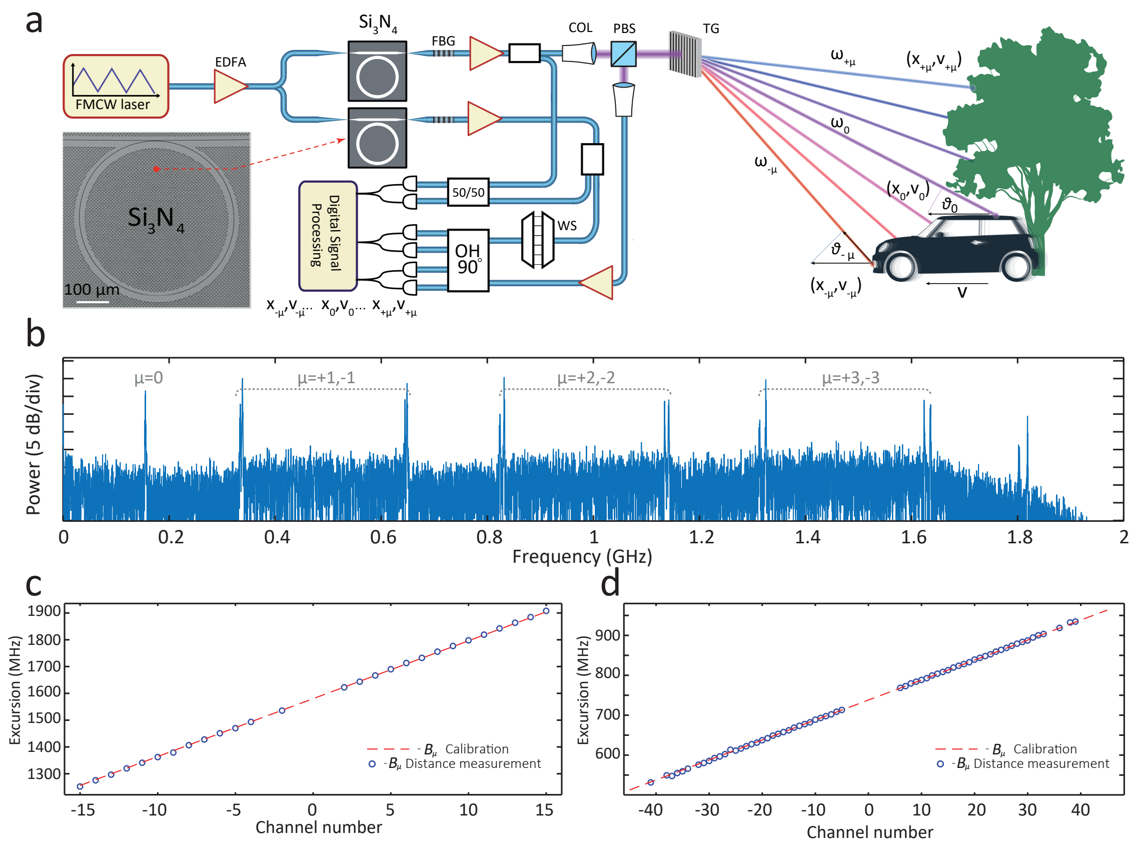

Supplementary Fig. 7. Excursion calibration. a) Architecture of the multiheterodyne parallel FMCW LiDAR. A pump laser with triangular frequency modulation drives two distinct optical microresonators with slightly different radii, which serve as signal and LO in the experiment. After pump filtering (FBG) and amplification (EDFA), the signal comb is spatially dispersed over the target area using diffractive optics. Each signal comb tooth μ represents an independent FMCW ranging channel measureing distance x_μ and velocity v_μ. All channels are simultaneously superimposed with the amplified LO comb on a coherent receiver. Waveshaper (WS) is used filter out excess ASE noise around the pump of the LO. Extra reference balanced photo-diode is used to measure frequency excursion. b) Interferogram of a LO and signal combs beating recorded over one period ($10\mu s$) on the reference photodiode with 1.6GHz bandwidth. Beatings of 6 channels ($\mu = \pm1, \pm3$) allow Δf_{rep} and B_μ to be retrieved and used for the processing of the measured data. c) Two frequency excursion measurements with 100 GHz chips. Red dashed line is an extrapolation of excursion obtained from the reference delayed heterodyne measurement. Blue circles correspond to an excursion obtained from a lidar distance measurement of a carton block, where all the distances are the same for distinct channels. d) Same as c), but with 35 GHz chips

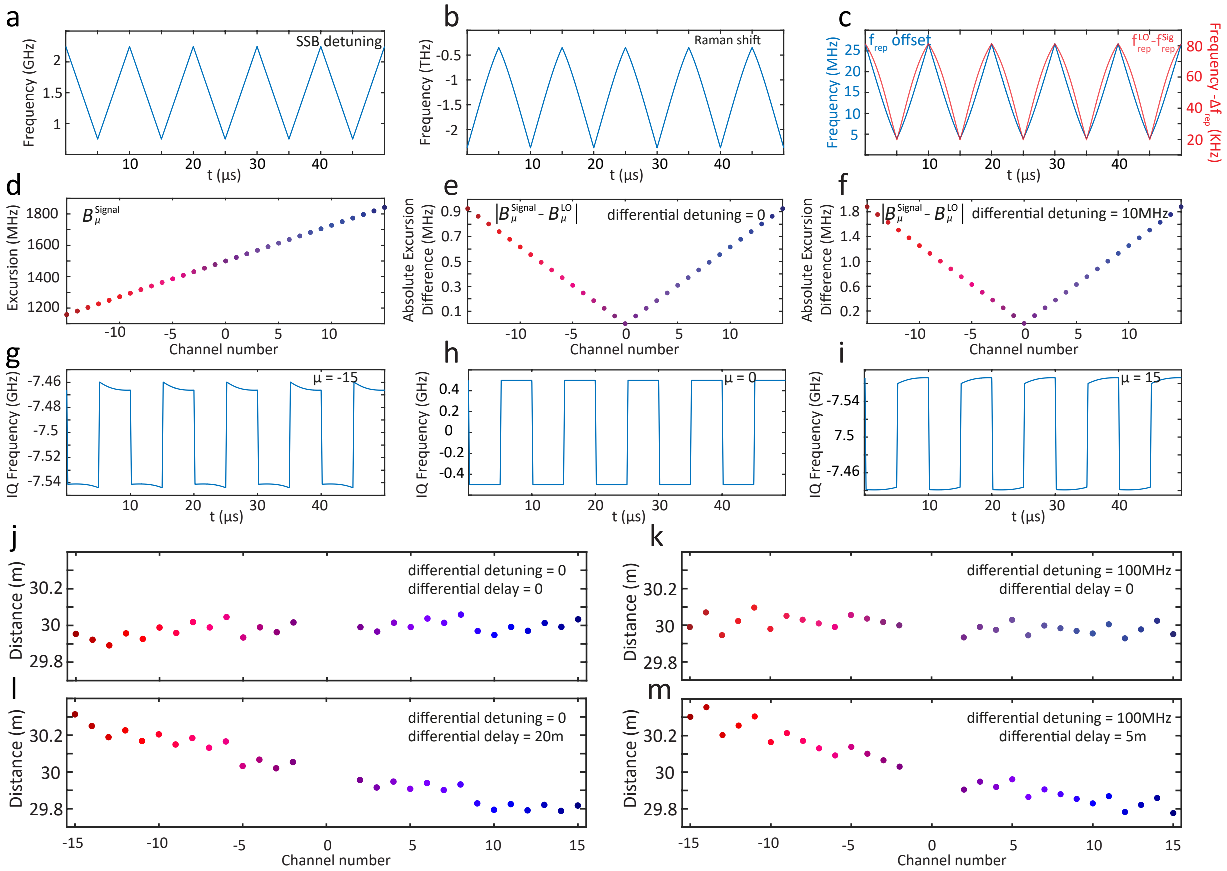

Supplementary Fig. 8. Raman nonlinearity impact in 100 GHz resonators. a) Simulation of a pump cavity detuning representing a triangular ramp. b) Simulation of a Raman shift experienced by a Soliton while being frequency swept. c) Blue - f_{rep} offset from a 100GHz caused by Raman nonlinearities. Red - Δf_{rep} offset from a 500 MHz (100 and 100.5 GHz free spectral ranges were used in this simulation) for the two solitons while being frequency swept due to Raman nonlinearities. d) Simulated frequency excursion for 100 and 100.5 GHz resonators due to Raman effect. e,f) Simulated absolute frequency excursion difference considering soliton-cavity detuning to be the same for two resonators and have 10 MHz difference correspondingly. g,h,i) Simulated time-frequency maps obtained from a beating of signal and LO combs. Difference in excursion due to Raman effect results in deformed trapezoidal traces. Channels $\mu = -15, 0, +15$ correspondingly. j,k,l,m) Simulated distance measurements calculated from time-frequency maps above. The term differential delay stands for the difference between optical delay of signal and LO for the real measurement on one side and optical delay of signal and LO for the reference calibration measurement on the other side.

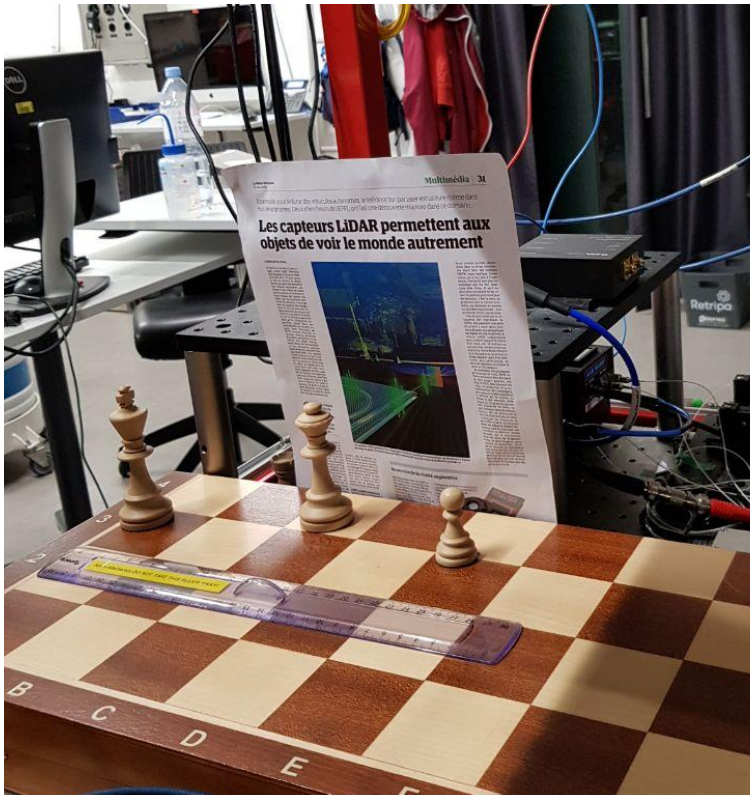

Supplementary Fig. 9. **Photograph of the imaged scene.** Pawn, Queen and King chess figures used for the lidar distance measurement.

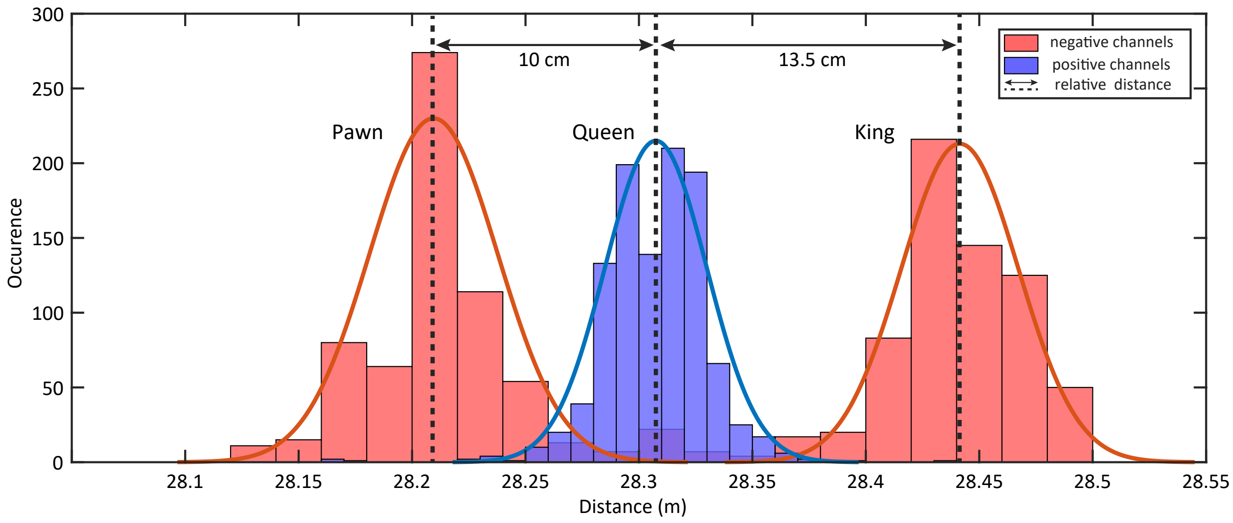

Supplementary Fig. 10. **Chess ranging measurement relative accuracy.** Measured relative distance for chess figures ranging experiment. Red histogram corresponds to the pixels in negative channels that illuminated Pawn and King, while blue histogram corresponds to Queen illuminated by positive channels. Histograms depict 136*28 measured pixels, where one line of 28 pixels was acquired during 10 μs time.

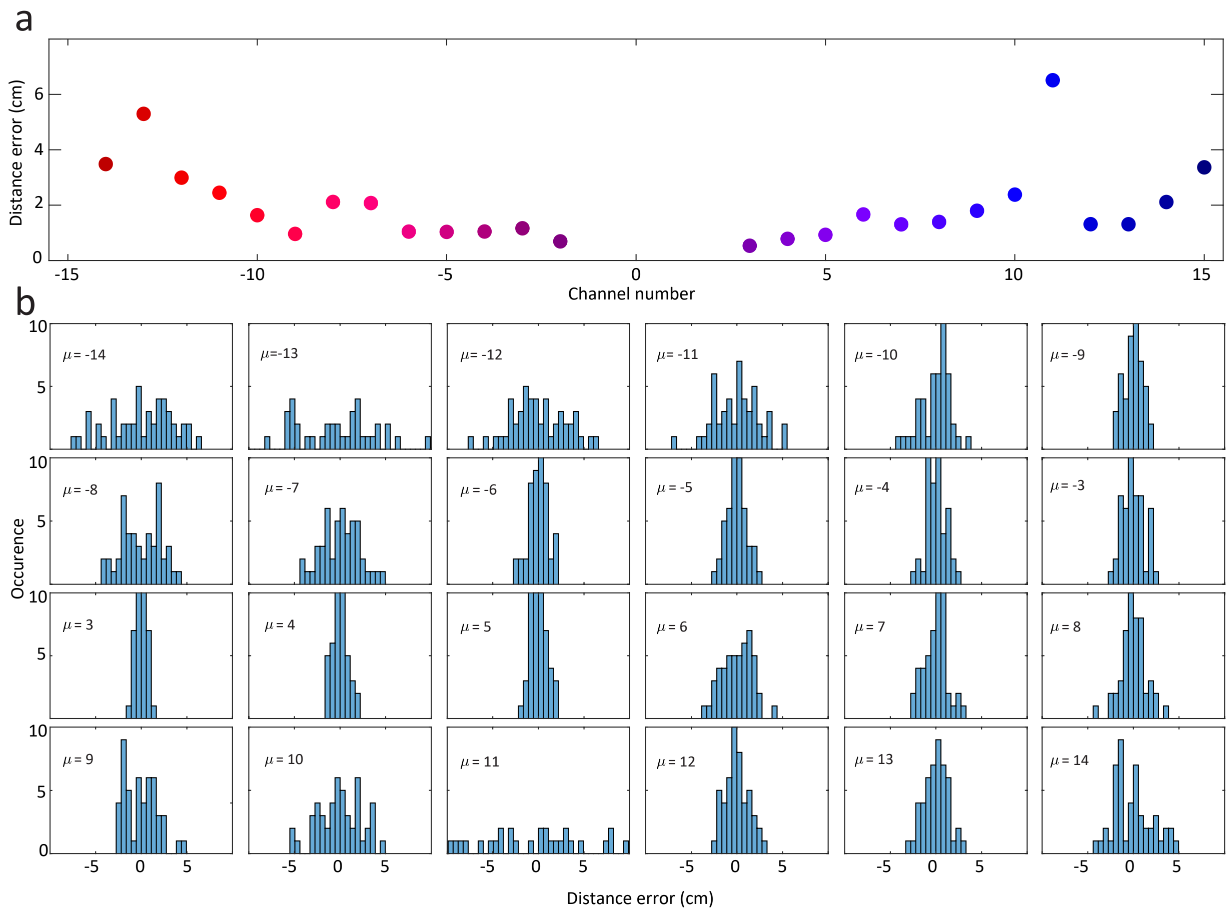

Supplementary Fig. 11. **Distance precision of the Dual-Comb LiDAR.** a) Channel-dependent distance precision. b) Histograms obtained during 49 distance measurements of the static object.

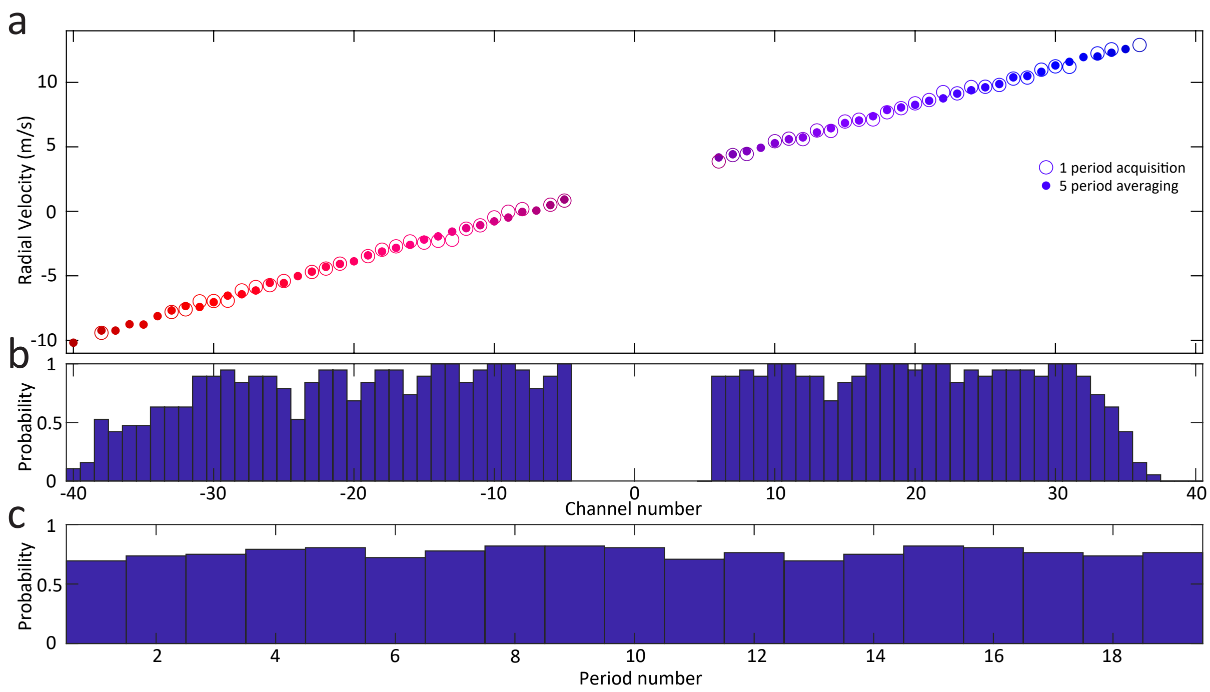

Supplementary Fig. 12. **Velocimetry measurements statistics.** a) Multichannel velocity measurement for the flywheel rotating at around 162 Hz for a single 10 μs scan (open circles) and five frame stacking (filled circles). b) Probability of a pixel to be detected in channel μ. Statistics are obtained over 19 periods measurement time ($190\mu s$). The roll-off at large distance from the pump laser originates from the limited optical amplifier bandwidth. c) Probability of detection over a given time frame ($10\mu s$). The probability is calculated as number of total pixels detected in that time frame divided by 81 ($\mu = -40, +40$)).

REFERENCES

---

[1] Liu, J. *et al.* Ultralow-power chip-based soliton microcombs for photonic integration. *Optica* **5**, 1347 (2018). URL https://doi.org/10.1364/OPTICA.5.001347. 1805.00069.

[2] Herr, T. *et al.* Temporal solitons in optical microresonators. *Nature Photonics* **8**, 145–152 (2014). URL https://doi.org/10.1038/nphoton.2013.343.

[3] Guo, H. *et al.* Universal dynamics and deterministic switching of dissipative kerr solitons in optical microresonators. *Nature Physics* **13**, 94–102 (2017). URL https://doi.org/10.1038/nphys3893. 1601.05036.

[4] Karpov, M. *et al.* Dynamics of soliton crystals in optical microresonators. *Nature Physics* **15**, 1071–1077 (2019). URL https://doi.org/10.1038/s41567-019-0635-0.

[5] Ahn, T.-J. & Kim, D. Y. Analysis of nonlinear frequency sweep in high-speed tunable laser sources using a self-homodyne measurement and hilbert transformation. *Applied optics* **46**, 2394–2400 (2007). URL https://doi.org/10.1364/AO.46.002394.

[6] Riemensberger, J. *et al.* Massively parallel coherent laser ranging using a soliton microcomb. *Nature* **581**, 164–170 (2020). URL https://doi.org/10.1038/s41586-020-2239-3.

[7] Gao, S., O'Sullivan, M. & Hui, R. Complex-optical-field lidar system for range and vector velocity measurement. *Optics express* **20**, 25867–25875 (2012). URL https://doi.org/10.1364/OE.20.025867.

[8] Hodgkinson, T., Harmon, R. & Smith, D. Demodulation of optical dpsk using in-phase and quadrature detection. *Electronics Letters* **21**, 867–868 (1985). URL https://doi.org/10.1049/el:19850612.

[9] Davis, A., Pettitt, M., King, J. & Wright, S. Phase diversity techniques for coherent optical receivers. *Journal of lightwave technology* **5**, 561–572 (1987). URL https://doi.org/10.1109/JLT.1987.1075539.

[10] Derr, F. Optical qpsk transmission system with novel digital receiver concept. *Electronics Letters* **27**, 2177–2179 (1991). URL https://doi.org/10.1049/el:19911347.

[11] Ellingson, S. Correcting iq imbalance in direct conversion receivers. *Argus Technical and Scientific Documents* (2003). URL https://www.faculty.ece.vt.edu/swe/argus/iqbal.pdf.

[12] Feneyrou, P. *et al.* Frequency-modulated multifunction lidar for anemometry, range finding, and velocimetry–1. theory and signal processing. *Applied optics* **56**, 9663–9675 (2017). URL https://doi.org/10.1364/AO.56.009663.

[13] Frigo, M. & Johnson, S. G. Fftw: An adaptive software architecture for the fft. In *Proceedings of the 1998 IEEE International Conference on Acoustics, Speech and Signal Processing, ICASSP'98 (Cat. No. 98CH36181)*, vol. 3, 1381–1384 (IEEE, 1998).

[14] Karpov, M. *et al.* Raman self-frequency shift of dissipative kerr solitons in an optical microresonator. *Physical Review Letters* **116**, 1–5 (2016). URL http://link.aps.org/doi/10.1103/PhysRevLett.116.103902.

[15] Yi, X., Yang, Q.-F., Yang, K. Y. & Vahala, K. Theory and measurement of the soliton self-frequency shift and efficiency in optical microcavities. *Optics letters* **41**, 3419–3422 (2016). URL https://doi.org/10.1364/OL.41.003419.

[16] Brasch, V. *et al.* Photonic chip–based optical frequency comb using soliton cherenkov radiation. *Science* **351**, 357–360 (2016). URL https://doi.org/10.1126/science.aad4811.

[17] Yi, X. *et al.* Single-mode dispersive waves and soliton microcomb dynamics. *Nature communications* **8**, 1–9 (2017). URL https://doi.org/10.1038/ncomms14869.

[18] Xie, X. *et al.* Phase noise characterization of sub-hertz linewidth lasers via digital cross correlation. *Optics letters* **42**, 1217–1220 (2017). URL https://doi.org/10.1364/OL.42.001217.

[19] Liu, J. *et al.* Monolithic piezoelectric control of soliton microcombs. *Nature* **583**, 385–390 (2020). URL https://doi.org/10.1038/s41586-020-2465-8.

[20] Kuse, N., Tetsumoto, T., Navickaite, G., Geiselmann, M. & Fermann, M. E. Continuous scanning of a dissipative kerr-microresonator soliton comb for broadband, high-resolution spectroscopy. *Optics Letters* **45**, 927–930 (2020). URL https://doi.org/10.1364/OL.383036.

[21] Baney, D. M., Gallion, P. & Tucker, R. S. Theory and measurement techniques for the noise figure of optical amplifiers. *Optical fiber technology* **6**, 122–154 (2000). URL https://doi.org/10.1006/ofte.2000.0327.

[22] Newbury, N. R., Coddington, I. & Swann, W. Sensitivity of coherent dual-comb spectroscopy. *Optics express* **18**, 7929–7945 (2010). URL https://doi.org/10.1364/OE.18.007929.
